# Laser Processed Antimicrobial Nanocomposite Based on Polyaniline Grafted Lignin Loaded with Gentamicin-Functionalized Magnetite

**DOI:** 10.3390/polym11020283

**Published:** 2019-02-07

**Authors:** Anita Ioana Visan, Gianina Popescu-Pelin, Oana Gherasim, Valentina Grumezescu, Marcela Socol, Irina Zgura, Camelia Florica, Roxana C. Popescu, Diana Savu, Alina Maria Holban, Rodica Cristescu, Consuela E. Matei, Gabriel Socol

**Affiliations:** 1 National Institute for Lasers, Plasma and Radiation Physics, 077125 Magurele, Ilfov, Romania; gianina.popescu@inflpr.ro (G.P.-P.); fufa.oana@inflpr.ro (O.G.); valentina.grumezescu@inflpr.ro (V.G.); rodica.cristescu@inflpr.ro (R.C.); consuela.matei@inflpr.ro (C.E.M.); 2 Department of Science and Engineering of Oxide Materials and Nanomaterials, Faculty of Applied Chemistry and Materials Science, University Politehnica of Bucharest, 011061 Bucharest, Romania; roxana.popescu@nipne.ro; 3 National Institute of Materials Physics, 077125 Magurele, Ilfov, Romania; marcela.socol@infim.ro (M.S.); irina.zgura@infim.ro (I.Z.); camelia.florica@infim.ro (C.F.); 4 Life and Environmental Physics Department, Horia Hulubei National Institute of Physics and Nuclear Engineering, 077125 Magurele, Ilfov, Romania; dsavu@nipne.ro; 5 Microbiology & Immunology Department, Faculty of Biology, University of Bucharest, 060101 Bucharest, Romania; alina_m_h@yahoo.com

**Keywords:** conductive polymers, magnetic nanoparticles, Gentamicin, matrix-assisted pulsed laser evaporation

## Abstract

Composite thin coatings of conductive polymer (polyaniline grafted lignin, PANI-LIG) embedded with aminoglycoside Gentamicin sulfate (GS) or magnetite nanoparticles loaded with GS (Fe_3_O_4_@GS) were deposited by the matrix-assisted pulsed laser evaporation (MAPLE) technique. The aim was to obtain such nanostructured coatings for titanium-based biomedical surfaces, which would induce multi-functional properties to implantable devices, such as the controlled release of the therapeutically active substance under the action of a magnetic and/or electric field. Thus, the unaltered laser transfer of the initial biomaterials was reported, and the deposited thin coatings exhibited an appropriate nanostructured surface, suitable for bone-related applications. The laser processing of PANI-LIG materials had a meaningful impact on the composites’ wettability, since the contact angle values corresponding to the composite laser processed materials decreased in comparison with pristine conductive polymer coatings, indicating more hydrophilic surfaces. The corrosion resistant structures exhibited significant antimicrobial activity against *Escherichia coli*, *Staphylococcus aureus*, and *Candida albicans* strains. In vitro cytotoxicity studies demonstrated that the PANI-LIG-modified titanium substrates can allow growth of bone-like cells. These results encourage further assessment of this type of biomaterial for their application in controlled drug release at implantation sites by external activation.

## 1. Introduction

The laser processing of antimicrobial composite materials with both electrical and magnetic properties, in the form of thin coatings, remains a highly topical and challenging subject of modern healthcare-related applications. Being considered a multivalent class of biomaterials, this type of smart surface has lately received prodigious attention from the international scientific community because of their great implication in both fundamental studies and practical biomedical applications [1,2].

In this context, composite coatings based on an intrinsic conductive polymer (polyaniline grafted lignin, PANI-LIG), embedded with magnetite nanoparticles (Fe_3_O_4_) and loaded with aminoglycoside antibiotic molecules (Gentamicin sulfate, GS), were deposited by the matrix assisted pulsed laser evaporation (MAPLE) technique, to examine their potential as electrically and magnetically controlled drug delivery platforms. The effectiveness of the selected laser processing method is dictated by the patterning facility for depositing on different geometric shapes of substrates in a highly uniform manner [3], the possibility to obtain multilayers or composite coatings [4], and a convenient transfer of a wide range of biomaterials [5,6]. Another important advantage of the MAPLE process consists in the elimination of major sources of contamination, proving to be a non-contact processing method suitable for specific medical applications [7]. An exhaustive comparison of MAPLE and other techniques used for growing thin films of polymers and biological materials was published by Chrisey et al. in [8].

Among conductive polymers, polyaniline (PANI) has attracted great interest thanks to its very good electrical and optical properties, environmental stability, facile synthesis, relatively low cost, tailorable conductivity, and ability to be modified in terms of porosity, biocompatibility, and biodegradability [9]. Furthermore, by grafting lignin in the PANI powder the tensile strength and bulk modulus of the basic polymer is improved. Further, the PANI-LIG composite is protected against oxidative degradation under UV light or elevated temperature [10]. However, the principal disadvantage of PANI is related to its poor processability. An efficient method for improving it is the inclusion within composite materials, thus, achieving a good balance between electrical and mechanical properties [11].

As well, the combination of conductive polymers with magnetic nanoparticles is an important alternative for processing new composite coatings for biomedical applications, such as drug delivery [12], cell imaging [13], diagnosis, and therapy [14,15].

Furthermore, the magnetic materials have great potential for simultaneous detection and imaging applicability, to be used as *T*_2_ contrast agents [16] or in magnetic hyperthermia [17] and drug delivery systems [18], which allows for accurate diagnosis and improved cancer treatment [19,20,21,22,23].

Moreover, it was proved that magnetic nanomaterials, especially nanosized or nanostructured systems and devices based on magnetite (Fe_3_O_4_), can enhance the therapeutic efficiency of medicative treatments by enabling the specific drug encapsulation and its further targeting ability for certain organs or injured anatomical areas inside the human body, and, thus, avoid drug-related toxicity with respect to healthy tissues [24].

Dielectric and electromagnetic materials interact in different ways with electromagnetic radiation, but the final mechanism resides in the transformation of the energy of the incident wave into heat. Depending on the application, it is necessary to precisely control the physical properties of the material in terms of permittivity, magnetic permeability and conductivity and to know their variations with the frequency, but at the same time to maintain specific weights and mechanical features. For these cases, intrinsically conducting polymers, such as polyaniline in particular, is a credible choice, especially given that its electromagnetic properties can be modified by the addition of inorganic fillers. Magnetite is a good candidate for this purpose, due to its good magnetic and electrical properties and very high saturation magnetization. Belaabed et al. studied the electromagnetic properties and absorbing behaviors of hybrid organic/inorganic materials providing evidence of how synthesis parameters, such as the amount and particle size of PANI and used Fe_3_O_4_, significantly affect the morphology, conductivity, and microwave absorption properties of the final materials [25].

In this context, we combined the conductive polymer (PANI-LIG) with magnetic nanoparticles loaded with Gentamicin sulfate (Fe_3_O_4_@GS) to increase the antimicrobial applications of the drug by inhibiting the adherence and suppressing the development of microbial biofilm [26,27]. Further, we sought to potentiate the therapeutic effects of the antibiotic [28].

Within our study, we selected Gentamicin as the model drug, the bactericidal aminoglycoside antibiotic being commercially available as Gentamicin sulfate (GS), and being acknowledged for its intrinsic efficiency in the treatment of various bacterial infections (e.g., osteomyelitis [29,30]). The chosen drug meets all requirements of the above-mentioned controlled delivery application, presenting activity against aerobic bacteria by blocking the synthesis of bacterial proteins [31].

Recently, Gentamicin has been reported to cause less resistance among pathogenic species, when compared to other antibiotics (e.g., Spectinomycin, Tetracycline, Ampicillin, Carbenicillin, Neomycin, and Sulfamethoxazole) [32]. We chose three microbial model strains, to evaluate the antimicrobial efficiency of the obtained nanocoatings, namely a Gram-positive bacterium, *Staphylococcus aureus* ATCC 25923, a Gram-negative bacterium, *Escherichia coli* ATCC 25922, and a yeast model, *Candida albicans* ATCC 10231 [32].

Although it shows sensitivity in vitro [33], the oral administration of Gentamicin is inefficient due to poor distribution in the intestinal mucosa, which reduces pathogens contact and elimination [33]. Thus, a specific drug delivery system of Gentamicin administration could increase efficacy and gastrointestinal transit in time and reduce possible unwanted adverse effects [33]. In our particular case, the porous structure of PANI may facilitate the successful adsorption of GS molecules within the polymer matrix.

Within this study, we evaluated the potential use of laser-synthesized composite coatings based on polyaniline grafted lignin and magnetite nanostructures embedded with Gentamicin sulfate as potential controlled drug release systems, with electric or magnetic external field activation. We report on the successful MAPLE transfer of antimicrobial composites consisting of electrically-responsive polymer and magnetically-active nanoparticles with tunable biomedical-related functionality.

## 2. Materials and Methods

### 2.1. Materials

All chemicals and biological materials employed in our synthesis and protocols were commercially available and guaranteed by their producer. Heptahydrate ferrous sulfate (FeSO_4_·7H_2_O), anhydrous ferric chloride (FeCl_3_), ammonia solution (NH_3_, 25%), polyaniline (emeraldine salt) short chain grafted to lignin powder (Mw ~50,000 g/mol), and Gentamicin sulfate (GS) were purchased from Sigma-Aldrich (Darmstadt, Germany). All chemicals used to synthesize the composite coatings were of analytical purity and used with no further purification. The solvent used for the preparation of MAPLE targets was dimethyl sulfoxide (DMSO) that was acquired from Merck (Merck, Darmstadt, Germany).

Some of the products required for biological investigations, such as paraformaldehyde, hexamethyldisilazane (HMDS) and non-essential amino-acids (NEAA), were also purchased from Sigma-Aldrich. Others, such as phosphate buffered saline (PBS), fetal bovine serum (FBS), Eagle’s Minimum Essential Medium (MEM), L-glutamine, and penicillin/streptomycin mixture (P/S), were procured from Biochrom Ltd. (Merck Milipore, Darmstadt, Germany). For the cellular viability evaluation, we used the MTS assay kit (CellTiter96^®^ Aqueous One Solution Cell Proliferation Assay) that was provided from Promega (Fitchburg, WI, USA). On the other hand, Texas Red^TM^–phalloidin solution—and Hoechst^®^ 33,342 stains used for the immunofluorescence studies were acquired from Invitrogen (Thermo Fisher Scientific, Waltham, MA, USA), while the human-derived osteoblasts-like cells (MG-63) and the microbial pathogens, namely *Staphylococcus aureus* (*S. aureus*, ATCC 25923), *Escherichia coli* (*E. coli*, ATCC 25922), and *Candida albicans* (*C. albicans*, ATCC 10231) were purchased from American Type Culture Collection (ATCC^®^, Manassas, VA, USA).

### 2.2. Synthesis of Fe_3_O_4_@GS Nanoparticles

The antibiotic-functionalized magnetite nanoparticles (Fe_3_O_4_@GS) were synthesized by the wet chemical co-precipitation method, according to our optimized protocol described in [34] and [35].

The metallic precursors (heptahydrate ferrous sulfate and anhydrous ferric chloride) were dissolved in ultrapure water. The resulted solution was dropwise added into an ammonia aqueous solution containing Gentamicin sulfate [36] and maintained under continuous stirring. The color of the resulted mixture gradually changed to black, as the metallic particles formed during the reaction. The collected precipitate was washed with ultrapure water and then dried at room temperature.

### 2.3. MAPLE Synthesis of Composite Coatings

To obtain the solid MAPLE targets, magnetite-free (with or without antibiotic addition) and antibiotic-functionalized magnetite embedded suspensions (PANI-LIG, PANI-LIG:GS and PANI-LIG:Fe_3_O_4_@GS powders suspended in DMSO as a 3% (*w*/*v*) solutions, respectively) were poured into a pre-cooled MAPLE target holder and subsequently immersed in liquid nitrogen for 15 min. The resulted frozen targets were kept at a temperature of ~173 K by introducing active liquid nitrogen cooling during the deposition.

The MAPLE experiments were carried out in a stainless steel reaction chamber at 5 Pa pressure, using a KrF* laser source (λ = 248 nm and τ_FWHM_ = 25 ns) model COMPexPro 205 (Lambda Physics-Coherent, Göttingen, Germany) that operated at a repetition rate of 20 Hz. A laser beam homogenizer was used to improve the energy distribution of the laser spot. Within our experiments, the deposition parameters were maintained constant, namely the target-substrate distance (5 cm), subsequent number of laser pulses (100,000), laser fluence (100 mJ/cm^2^), and laser spot area (33 mm^2^). During the deposition process, the frozen targets were rotated at a rate of 50 Hz, to avoid the target heating and drilling due to the multiple laser irradiations. In addition, to improve the uniformity of deposition, the substrates were continuously rotated at a rate of 50 Hz.

The simple and composite coatings were deposited onto grade 4 commercial titanium disks (12 mm in diameter and 0.2 mm thickness), transparent infrared (IR) (100) silicon slides and glass slides (10 × 10 mm^2^), depending on physico–chemical characterization or biological assays, respectively. All depositions were conducted at room temperature, and before the deposition experiments, all the substrates were subjected to a successive ultrasonic cleaning treatments with acetone, ethyl alcohol and deionized water for 15 min and further dried in a jet of high purity nitrogen. The as-deposited samples were denoted as PANI-LIG (pristine polymer samples), PANI-LIG:GS (composite antibiotic-loaded polymer samples). and PANI-LIG:Fe_3_O_4_@GS (composite polymer samples embedded with antibiotic-functionalized magnetite). For data comparison, a control set of coatings were prepared by dropcast on double side polished Si (100) and glass substrates, to evaluate the potential chemical changes occurred during the laser transfer.

### 2.4. Physico-Chemical Characterization

(i) X-ray diffraction (XRD) analysis was performed to investigate the crystalline nature of the deposited coatings. A Bruker D8 Advance diffractometer (Bruker AXS, Karlsruhe, Germany), equipped with a Cu target X-ray tube, in the parallel beam setting was used. In all cases, K*α* radiation from a Cu X-ray tube (run at 15 mA and 30 kV) was used. The samples were scanned in the Bragg angle 2*θ* range of 20 to 70°, with a step size of 0.04°, and 6 s per step.

(ii) Fourier transform infrared spectroscopy (FTIR) was performed for stoichiometry and chemical functions integrity investigation of the as-deposited coatings. A Shimadzu FTIR 8400 s spectrophotometer (Shimadzu Europa GmbH, Duisburg, Germany), operating in transmission mode in the range of 5000 to 500 cm^−1^, with a resolution of 4 cm^−1^ was used, and for each sample, a set of 50 individual scans was acquired.

(iii) Scanning electron microscopy (SEM) analysis revealed the morphological features of the deposited materials and it was performed on an ApreoS scanning electron microscope (Thermo Fisher Scientific, Waltham, MA, USA), using secondary electron beams with energies of 10 kV, on samples capped with a thin gold layer to diminish the accumulation of electric charges on the specimen surface.

(iv) Atomic force microscopy (AFM) was performed for topographical evaluation of the deposited coatings by using a MultiView 4000 Nanonics System (phase feedback, Nanonics Imaging Ltd., Jerusalem, Israel) over a scanned area of (40 × 40) μm^2^, in tapping mode.

(v) The static contact angle (CA) measurements are a representative measure for surface wettability and are often employed to evaluate coatings surface properties. The investigations were performed on a Drop Shape Analysis System, model DSA100 with DSA3^®^ software (Kruss GmbH, Hamburg, Germany), using water as a standard solution, at room temperature, by the goniometric method. In this context, 2 μL of water was dropped in a controlled manner on samples’ surface, maintaining constant the droplet volume and the dropping distance. Thus, the droplet image was recorded under a 2° angle with respect to the plan of the examined sample. To obtain the CA values, the acquired data were fitted using the second-degree polynomial equation, and the slope values corresponding to the tangent drawn by the drop were extracted [37]. The reported CA values are depicted as the average of two different measurements on the same sample.

(vi) Electrochemical investigations were performed with an OrigaStat 100 galvanostat–potentiostat system model from OrigaLys (Origalys ElectroChem SAS, Rillieux-la-Pape, France). The investigated samples, namely PANI-LIG:GS (simple antibiotic-embedded samples) and PANI-LIG:Fe_3_O_4_@GS (samples embedded with antibiotic-functionalized magnetite) were prepared for electrochemical tests by their immersion for 24 h in simulated body fluid (SBF), prepared after the Kokubo recipe [38]. Before starting the electrochemical measurements, the open circuit potential (OCP) reached a stable value, in all experimental setups.

We used a three-electrode measuring cell, in which the tested sample was the working electrode (with an exposed area of 0.8 cm^2^), while the Ag/AgCl and Pt electrodes represented the reference and counter electrodes, respectively. The data acquisition and processing were achieved with the OrigaMaster5 software, the scan rate being set at 1 mV/s and the working potential from −300 to +300 mV (vs. Ag/AgCl electrode). The AC potential and frequency applied to the electrochemical cell was of 0.01 V amplitude and 100,000–0.1 Hz, respectively. Forward, the current through the cell was measured. For data processing, Origin 6.0 (OriginLab, Northhampton, MA, USA) was used.

### 2.5. Biological Investigations

#### 2.5.1. In Vitro Cytotoxicity Assays

The in vitro cytotoxicity evaluation of the PANI-LIG-based thin coatings was done for osteoblast-like cells (MG-63) (ATCC, Manassas, VA, USA) [39] and [40]. Samples were sterilized by UV exposure during 1 h and washed with complete culture medium (Eagle’s MEM- Biochrom, supplemented with 10% FBS, 1% L-glutamine, 1% P/S, and 1% NEAA at 37 °C) during 10 min.

Cell stocks were cultured in Eagle’s MEM- Biochrom, supplemented with 10% FBS, 1% L-glutamine, 1% P/S, and 1% NEAA at 37 °C and kept in standard conditions of temperature and humidity (37 °C, 5% CO_2_) until they reached confluence. Afterwards, they were detached using 1% Trypsin, seeded at a density of 10,000 cells/sample onto the PANI-LIG substrates. These were further incubated in standard conditions, during 48 h, to examine the morphological behavior of the osteoblast-like MG-63 cells [41]. The metabolic activity of the cells after being in contact with the PANI-LIG substrates was measured using the MTS tetrazolium salt viability assay (CellTiter96^®^ Aqueous One Solution Cell Proliferation Assay) as in [42]. Results were presented as reported to control’s absorbance (Ti). An immunofluorescence assay was employed to reveal the morphology and integrity of the cytoskeleton of the cells after being in contact with PANI-LIG substrates. For this, Texas Red^TM^–phalloidin was used to mark the actin filaments, while Hoechst was used as counterstaining of nuclei. Fixing and staining of the samples for fluorescence microscopy was done as in [42]). The images were acquired using a BX41 fluorescence microscope from Olympus (New York, NY, USA). The SEM morphology investigations were performed to obtain complementary and more detailed information on the behavior of the MG-63 cells at interaction with the previously obtained thin coatings. Investigations were done after 48 h of incubation, and the substrates were cultured as for fluorescence microscopy investigations. The samples were fixed and prepared for SEM as in [42] and [43].

Micrographs were acquired using an FEI Inspect S microscope (FEI, Thermo Fisher Scientific, Hillsboro, OR, USA) operating at 20 kV acceleration voltage.

#### 2.5.2. Anti-Biofilm Assay

The anti-biofilm assays of the deposited thin coatings were performed according to ASTM E2180-07 (2012) standard by adjusting the protocol described in [44,45]. The anti-biofilm assays were performed against Gram-positive (*Staphylococcus aureus* ATCC 25923) and Gram-negative (*Escherichia coli* ATCC 15224) bacteria, and also against fungal strain (*Candida albicans* ATTC 10231). Glycerol stocks of these microbial strains were streaked on nutritive agar plates and colonies were allowed to grow at different for 24 h at 37 °C.

Before testing, all deposited coatings were sterilized by UV radiation exposure for 30 min. For assessing monospecific biofilms formation, 2 mL of liquid nutrient medium (nutrient broth) was added to the sterilized sample independently placed in 6-well plates containing either test (deposited samples) or control (uncoated) substrates. The microbial inoculum consisted of a volume of 20 μL of the microbial suspension prepared by fresh colonies (corresponding to 0.5 and 1 McFarland optical densities for bacterial and fungal strains, respectively) prepared in phosphate buffered saline (PBS) [46].

Biofilm formation on the materials was assessed after incubation for distinctive periods, by the viable cell count (VCC) method. Concretely, after the incubation period, the samples containing attached pathogenic organisms were gently washed with sterile physiological PBS and transferred into a new sterile tube, containing 1 mL of fresh and sterile nutrient broth and vigorously vortexed for 30 s to detach the biofilm-embedded cells. The obtained cellular suspensions (PBS suspensions containing detached bacterial and fungal cells, respectively) were then serially diluted, and each dilution was spot-inoculated onto nutritive agar, to achieve microbial growth and to estimate the number of colony forming units (CFU)/mL [46]. Experiments were performed in triplicate.

## 3. Results and Discussion

### 3.1. Physico–Chemical Characterization

#### 3.1.1. XRD Measurements

**Figure 1:**
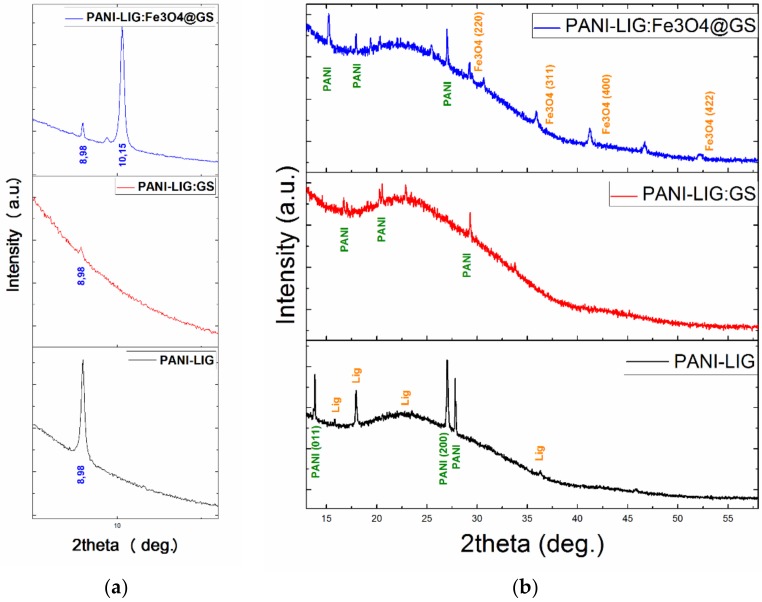
X-ray diffraction (XRD) patterns of polyaniline grafted lignin (PANI-LIG)-based coatings, corresponding to 7.5 to 13° (**a**) and 13 to 58° (**b**) diffraction angle regions, respectively.

Given the fact that some diffraction peaks corresponding to the pristine polymer were overshadowed by other maxima corresponding to the obtained composites, we considered providing a thorough overview with respect to the nature of the deposited coatings by including the collected XRD data within two distinctive plots. Therefore, the diffractograms presented in Figure 1 include an intimate and zoomed in view of the 7.5 to 13° diffraction region (Figure 1a), but also a general XRD pattern recorded in the 13 to 58° diffraction angles zone (Figure 1b).

The diffraction peaks observed in Figure 1 are found to be in agreement with the literature data regarding PANI-based coatings [47], indicating that all deposited coatings are poorly crystalline.

The principal diffraction peaks corresponding to the conductive polymer can be observed in all deposited samples, either pristine coating or coatings embedded with sole antibiotic or antibiotic-loaded magnetic nanoparticles. The identified diffraction maxima correspond to approximate 2θ diffraction angles of 8.98°, 10.15°, 13.83°, 15°, 17.94°, 20.36°, 25.45°, and 27°, respectively. The broad peak centered at 2θ ≈ 15° corresponds to the (011) diffraction plane assigned to basic polymer. The peaks located at ~21°, corresponding to (020) crystal plane of orthorhombic polymer in its emeraldine salt form, could be related to the chain repeated units and parallel periodicity [48]. The peak at ~26°, corresponding to the (200) diffraction plane, is due to the periodicity in the perpendicular direction of the conductive polymer chain [49]. The fairly strong intensities of the main peaks suggest a short-range order of the counter-ions along the lignin chain interposed with polymer salts [50]. Further, the broad embossment of the pattern with the absence of any sharp diffraction of the grafted component, indicate that lignin (which is a three-dimensional amorphous polymer consisting of methoxylated phenyl-propane structures) [51] decreases the relative degree of crystallinity in all deposited coatings.

The XRD pattern corresponding to the PANI-LIG materials embedded with drug-loaded Fe_3_O_4_ nanoparticles exhibits, in addition to sole polymer peaks, diffraction peaks that correspond to crystalline magnetite (marked in orange). The identified additional maxima present the following diffraction angle–crystal plane pairs: 30.36° – (220), 35.75° – (311), 43.52° – (400) and 53.95° – (422), which are attributed to diffraction planes of face-centered cubic Fe_3_O_4_ in its pure spinel form [51]. Changes in the intensity and position of the XRD peaks were observed due to the surface functionalization reaction, thereby indicating that ion migration occurred in the lattice of the metal oxides [52,53,54].

#### 3.1.2. FTIR Measurements

The compositional investigation of the MAPLE coatings, performed by the identification of the main infrared maxima corresponding to the materials considered within our study, indicates a successful and quasi-stoichiometric laser transfer.

**Figure 2:**
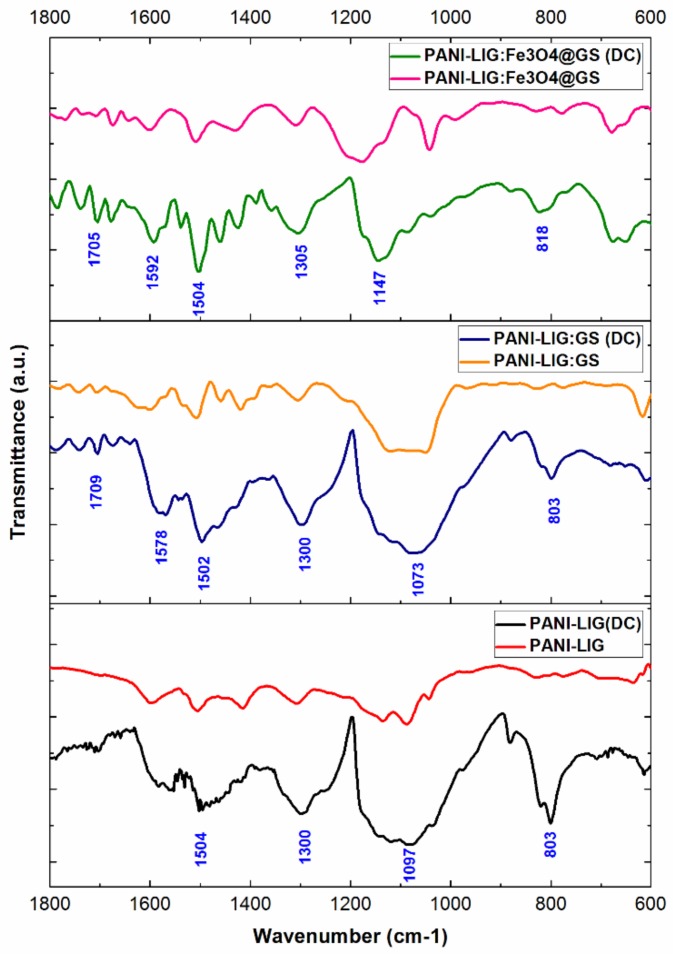
Fourier transform infrared spectroscopy (FTIR) spectra of PANI-LIG-based coatings.

For comparison reasons, we separately represented the infrared spectra of each MAPLE coating and compared it with the corresponding dropcast (DC) results (Figure 2). As a general remark, almost all principal vibrational peaks assigned to the conductive polymer are revealed in all dropcast and MAPLE coatings, with only a slight shift of the specific absorption maxima being observed in the case of MAPLE coatings, as well as a corresponding merging of infrared peaks into wide absorbance bands and a decrease in amplitude of the identified infrared maxima (suggesting the presence of thinner coatings).

In the case of pristine PANI grafted lignin coatings (Figure 2, bottom spectra), it can be observed that the infrared absorbance maxima of lignin gradually overlapped by those corresponding to the conductive polymer. Such a phenomenon is related to the grafting process and the formation of binary blends between PANI and LIG, due to the successful interaction of lignin with the conductive polyaniline through their hydroxyl and carbonyl groups. However, the MAPLE processed PANI-LIG coating presents similar infrared maxima as in the case of DC specimen, the identified absorption peaks being in good accordance with the results described in the literature for PANI-based materials [55,56].

The band identified at 1705 cm^−1^ may result from the superposition of e C=O and C=N (assigned to the quinoneimine group) stretching vibrations of PANI polymer. The adjacent low-intensity absorption bands (identified below 1700 cm^−1^ and above 1600 cm^−1^ wavenumber values) have specifically resulted from the stretching vibrations of carbonyl group within lignin. The characteristic vibrations identified at ~1590 cm^−1^ and ~1504 cm^−1^—resulted by the stretching modes of C=C functional group within quinoid (N=Q=N) and benzenoid (N=B=N) rings within PANI, respectively, and represent a clear signature of the polymer backbone [57]. The C=C vibration within the quinoid structure is also characteristic for lignin.

The low-intensity absorbance maximum observed within the previous values, concretely at ~1514 cm^−1^, is particularly assigned to C=C vibration of the aromatic lignin ring. Another absorbance maxima characteristic for lignin is identified at ~1460 cm^−1^ wavenumber and is the consequence of methoxy infrared vibration.

The peak at ~1300 cm^−1^ is due to the C–N strong stretching vibrations within aromatic amines in PANI [58].

The absorption maximum identified at ~1147 cm^−1^, of low intensity, may be specifically assigned to the C–H in-plane bending vibrations (corresponding to a 1,4-disubstituted aromatic ring of PANI). This particular band is also mentioned in the literature as the “electronic-like band signature” of polyaniline. The following infrared peaks (identified at ~1090 cm^−1^) may result from both organic compounds, as a consequence of superposed C–H bending (within PANI) and β-O-4 vibration (ether bond band of lignin).

The infrared band around ~818 cm^−1^ (indicating the head-to-tail coupling within polymer structure) is related to the out-of-plane bending mode of aromatic C–H groups attributed to 1,4-disubstituted aniline units [59].

In the case of antibiotic-loaded MAPLE samples (Figure 2, middle spectra), the incorporation of Gentamicin sulfate into the polymer structure caused a slight shift of the previously identified infrared maxima corresponding to PANI-LIG. In addition to the already mentioned absorbance maxima of pristine polymer, the presence of a distinct peak at 604 cm^−1^, which is particularly attributed to the stretching of SO_2_ band within Gentamicin, can be observed. In addition, the absorbance band identified at ~1097 cm^−1^ may result from the vibrations of HSO_4_^−^ group of the antibiotic. As well, we may consider that the infrared region in the range of 1110 to 1180 cm^−1^ contains absorbance maxima corresponding to both organic PANI and LIG (as discussed above) but also to Gentamicin sulfate (due to constituent C–H vibrations). The 1367 cm^−1^ absorbance peak can also correspond to both PANI and GS, following the overlapping of C–N modes from both compounds and C–O vibrations within the aminoglycoside. The overall shifting tendency of the infrared data corresponding to PANI-LIG:GS coatings may be explained by the weak electrostatic interaction between the secondary amines within polymer structure and the antibiotic hydroxyls.

As for the infrared data corresponding to PANI-LIG coatings embedded with antibiotic-functionalized magnetite nanoparticles (Figure 2, top spectra), the collected spectra show some additional peaks. The infrared maxima positioned at 584 cm^−1^ is the indication for SO_2_ band within Gentamicin sulfate, while the recorded shifted value (when compared to the previous data) might have resulted from the weak interactions established between antibiotic and hydroxylated magnetite. The presence of Fe–O bond is clearly evidenced at ~647 cm^−1^ wavenumber. On the other hand, the possible distinctive interactions between the amino functions of GS and the metallic ions within Fe_3_O_4_ might have determined the splitting of the Fe–O absorbance band (following potential lattice deformation processes) [60].

At this point, one can state that the MAPLE technique proved an effective strategy for the synthesis of PANI-LIG-based coatings, enabling the preservation of chemical functions for all considered materials and their quasi-stoichiometric transfer.

#### 3.1.3. SEM Examination

**Figure 3:**
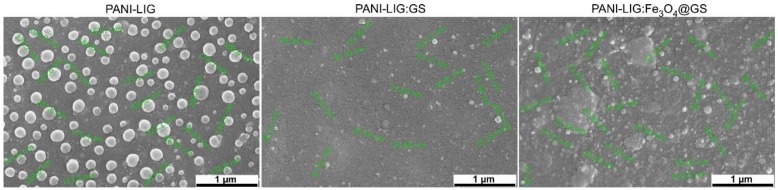
Scanning electron microscopy (SEM) micrographs of PANI-LIG-based coatings.

The SEM images of all MAPLE samples (Figure 3) reveals a predominant granular morphology, with particulate aggregates uniformly distributed onto the substrates.

It can be observed that all PANI-LIG-based coatings formed compact and uniform layers on the substrates, and it also covered them entirely. Instead, even though all granular and preferentially spherical shaped structures are nanosized, their dimensional range is related to the composition of the coatings, as follows: 100 to 300 nm for simple PANI-LIG samples, and 40 to 100 nm for both PANI-LIG:GS and PANI-LIG:Fe_3_O_4_@GS composites. It was observed that Gentamicin sulfate and magnetite compounds are uniformly spread into the PANI-LIG matrix, but after the surface functionalization reaction, both the size and shape grains altered. Because the formation of magnetite aggregates is directly related to their nanosized dimensions [61], magnetic-functionalized samples (Figure 3) revealed an increased density of granular structures within the entire polymer matrix. It can be observed that the surface morphologies become smoother and more compact as can also be observed from further AFM investigations (especially for antibiotic-loaded samples). In compliance with the literature data, the modified co-precipitation synthesis protocol rather enables the formation of quasi-spherical nanoparticles, with preferential core/shell structure [62,63]. By using a similar synthesis strategy, one may assume that, as in our case, the antibiotic molecules were organized as a thin layer onto the surface of the metallic iron oxide nanostructures. In addition, we presumed that, in the case of PANI-LIG:Fe_3_O_4_@SG samples, a protective antibiotic layer was formed on the metallic nanoparticles surface (Fe_3_O_4_), almost entirely dispersed on the surface of the coating. Thus, fewer surface-active sites for particle-particle interactions [52] remained. Based on this observation, one can conclude that those antibiotic-loaded structures, having a higher molecular weight than gentamicin, cover the polymer partially. Thus, a significant quantity of active sites for antibiotic–polymer interparticle interactions remained.

In addition to that, the crystalline nature of samples is corroborated by the SEM micrographs. The composite samples (PANI-LIG:GS and PANI-LIG:Fe_3_O_4_@GS) exhibit better cohesion and a higher aggregation of grains (resulting in a more amorphous structure of coatings) than the much-ordered alignment arrangement in the case of PANI-LIG. In addition, in good agreement with literature data, that antibiotic and magnetite presence may influence the basic polymer morphology, hydrophilicity and solubility [54]. The SEM images show that the simple conductive polymer grains are distinguishable in nature and loosely packed, most probably as a result of PANI-LIG hydrophobicity [54]. SEM investigations confirmed that the granular structures with specific irregular and randomly shaped particulate morphology are present in composite coatings. Morphological analysis of the pristine conductive polymer showed the presence of globules, which are absent in both PANI-LIG:GS and PANI-LIG:Fe_3_O_4_@GS coatings.

At the same time, we know that the solvent type may influence the surface morphology. Since PANI is partially insoluble in DMSO and was deposited from a frozen suspension, one can assume the intense structural reorganization of the organic polymer after laser transfer [54].

#### 3.1.4. AFM Investigations

**Figure 4:**
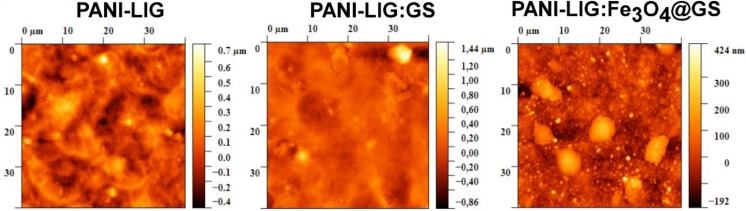
Atomic force microscopy (AFM) images of PANI-LIG-based coatings.

All the surfaces display similar topographies (Figure 4) with smooth surfaces and uniformly distributed nanometric grains covering the entire exposed area of the sample. The AFM measurements at 40 × 40 μm^2^ area showed a surface roughness of about 134 nm for PANI-LIG coatings, 137 nm for antibiotic–polymer coatings, and 80 nm for magnetic–antibiotic functionalized samples, respectively.

In the MAPLE deposited coatings, the antibiotic samples exhibit larger superficial particulate structures with higher roughness values that revealed a greater bacterial inhibition zone. This zone might be considered as an antibacterial activity due to the presence of Gentamicin sulfate. The lower roughness values obtained for magnetic-antibiotic–polymer samples are assumed to be from the good inorganic clusters incorporation and uniform distribution into the polymer matrix, confirming the SEM results.

Quantitative measurements of surface roughness are revealed in Table 1. Based on these results, regardless of the composition of the coatings, the skewness (*R*_sk_) parameter indicates a predominant compact flat surface with peak-like topography exhibiting positive values lower than 1.6.

In the case of samples with antibiotic, the substantial contrast between the RMS and *R*_sk_ parameters is attributable to the reduced presence of peaks on the surface and leads to a homogenous feature of height distribution [64,65] confirming, thus, our previous assumption regarding the polymer–antibiotic interactions and the composite structural reorganization after the MAPLE process. The magnetic–antibiotic samples present larger inorganic-based aggregates due to magnetite incorporation into the polymer matrix but with reduced skewness values.

As a further matter, the kurtosis (*R*_ku_) parameters (with values around 8 for simple and magnetic–antibiotic functionalized samples and around 13 for antibiotic samples) supports the skewness observations indicating, once again, that the MAPLE processing method leads to predicted smooth topographies, as are desired for medical applications.

#### 3.1.5. Wettability Measurements

The surface wettability investigation involves the contact established between a liquid (water) and a solid (either uncoated or MAPLE deposited coatings). The contact angle (CA) is a reliable parameter in biomaterials characterization since modifying and/or controlling the wettability behavior is of great importance for cell adhesion and proliferation.

**Figure 5:**
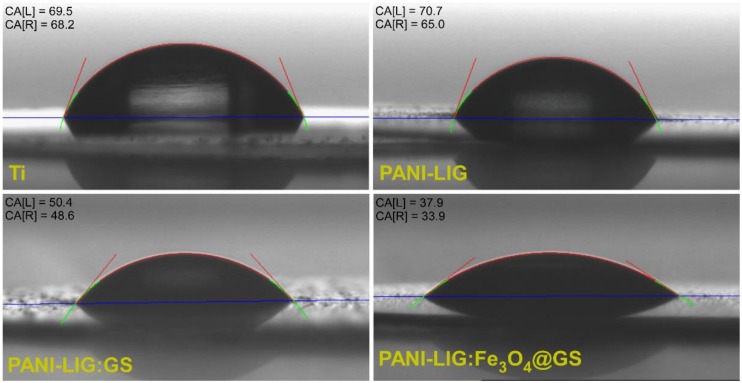
Representative images of water droplets on bare Ti and Ti with PANI-LIG based coatings deposited by matrix-assisted pulsed laser evaporation (MAPLE).

CA average values are found in Table 2 meanwhile typical CA images for water drop evolution in time for PANI-LIG, antibiotic–polymer and magnetic functionalized antibiotic–polymer coatings are reported in Figure 5.

As a general remark, it can be observed that simple conductive polymer coatings exhibit an intrinsically hydrophilic behavior with a contact angle value of 64 ± 3 (it resists water molecules). The tendency is to leave the aqueous solution or to associate its several molecules by hydrophobic interactions so that its contact with water molecules is minimal (by the formation of micelles aggregates). Moreover, it is known that the variation of the pristine PANI-LIG hydrophobicity affects both cells attachment and spread [66].

Nevertheless, laser processing as thin coatings has a significant impact on composites’ wettability, and one observes that CA values decrease in comparison with a pristine conductive polymer, indicating more hydrophilic surfaces, which results in an increase of water permeability of the composite coatings. In addition, all wettability measurements revealed a slight decreasing tendency of CA values, when compared to bare Ti substrate (69.5 ± 4.5).

A lower contact angle was evidenced for antibiotic-embedded polymer coatings (49.5 ± 0.9), suggesting that water droplets placed on the composite coatings surface immediately penetrate into the grains. This behavior can be explained by the PANI-LIG porous nature, possessing high surface energy that causes the water to seep into the voids of the structures [67]. In general, the incorporation of Gentamicin sulfate has led to an increase in hydrophilicity, as compared to basic polymer coatings.

One observes that earlier surface topographic results (relevant for human osteoblast differentiation and proliferation [68]) are entirely supported by contact angle measurements (which influence both endothelial cell [69] and human fibroblast attachment [70].

Optimal wettability values were obtained for PANI-LIG:Fe_3_O_4_@GS 35.9 ± 2.0°, the results being in good agreement with AFM observations, which obtained lower roughness values. The approximately flat surface of the sample permitted the water drop dripping, thus, justifying the small CA values obtained. Moreover, from long-term studies concerning the drop evolution in time (Table 2), one observes that all studied samples revealed a rather hydrophilic behavior.

As a general remark, the successful incorporation and distribution either of individual magnetite and/or antibiotic (as resulted from SEM and AFM investigations) has significantly improved the composite coating’s wettability behavior, indicating the possibility to adjust the contact angle values of chosen biomaterials to biological requirements.

#### 3.1.6. Electrochemical Evaluation

**Figure 6:**
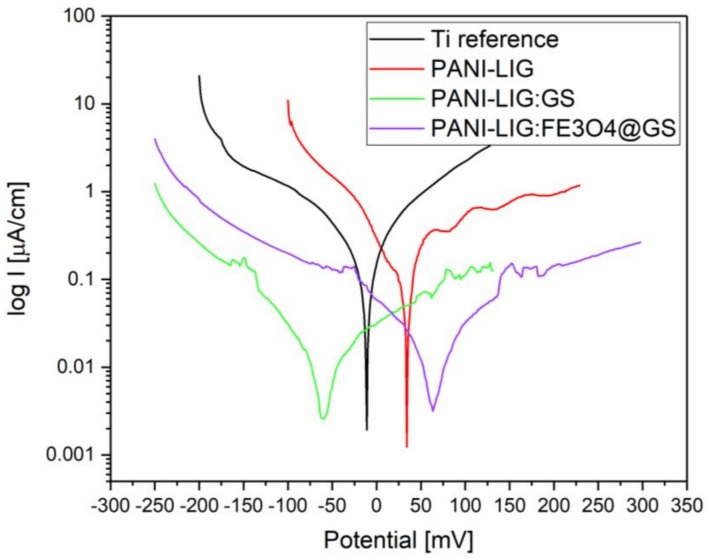
Tafel representation of polarization resistance obtained after immersion in simulated body fluid (SBF) (pH = 7.4).

The antioxidant capacity of polymeric coatings (ability to be oxidized and reduced, including the scavenging of free radicals) may offer protection against various diseases, such as cardiovascular diseases, osteomyelitis or cancer [71], in particular, in tissues suffering from oxidative stress, where the ability to reduce reactive radical species excessive levels is desirable [72]. Significant results for electrochemical tests are shown in Figure 6 and Table 3. The corrosion protection of the coatings can be observed from the values of *E*_corr_, *R*_p_ (calculated from the Tafel plots), *I*_corr_ (determined by the intersection of the linear portions of the anodic and cathode curves), and CR, listed in Table 3; and generally, a higher *E*_Corr_ and a lower *I*_corr_ indicate a better/good corrosion protection.

Polarization resistance values obtained from electrochemical impedance spectroscopy data and from Tafel curves are congruent series, supporting a decrease of corrosion current in PAN-LIG samples.

As a general remark, corrosion potential differences are small because the material exposed to corrosion remains the substrate (titanium), and also the measured corrosion current refers mainly to the support material (titanium coated naturally with TiO_2_) because the electrolyte diffuses through the deposited polymer layer. However, when compared to reference Ti substrate, the enhanced corrosion behavior of PANI-LIG coatings may be assigned to limited diffusion of electrochemically-active species to the substrate. As well, compounds with lower formal potentials are considered stronger reducing agents, and in this observation, are more powerful antioxidants [73].

One can observe that compared to a bare substrate, the current density *I*_corr_ dropped ~2.31 and 1.39 times, while the corresponding Ecorr increased ~3.7and 4.67 times in the case of PANI, PANI-GS, PANI-LIG:Fe_3_O_4_@GS coatings, respectively.

The significantly lower corrosion rate of lignin covered samples (below 47% relative to the reference) is interpreted as a corrosion protection effect, obtained by limiting the diffusion of the support material.

Furthermore, it is known that polyaniline is usually electroactive only under acidic conditions (pH less than 4 [1]), while in the presence of lignin [74]) and can produce a grafted form of the polymer that is electroactive also at neutral pH. Furthermore, the simple lignin film is not presenting a redox process in the cyclic voltammetry assays [75], such as the oxi-reduction processes which appear in the PANI-LIG coatings and suggest the lignin and polymer interaction.

In the case of PANI-LIG:GS coatings, we observed quick corrosion in the early stage. We presume, since the coatings are thick and stable and act as a barrier, that the compound molecules must travel a long distance from the coating to the medium. Consequently, the OCP is much slower (56 mV). Undoubtedly, as the antibiotic was added, the coating protection ability was enhanced. Comparable *R*_p_ values were reported for the PANI LIG:GS coatings led us to assume that the initial electrochemical reactions between the PANI-LIG:GS coatings and the electrolyte happened. This may cause the partial release of the incorporated compounds resulting in limited areas exposure of the substrate to new interactions with the electrolyte. However, such potential mechanism did not severely affect the titanium, since it is naturally protected by the native oxide layer [42].

The mechanism of the enhanced corrosion protection effect of the composite coatings, both the simple antibiotic functionalized, and magnetic nanoparticles embedded drug might be a result of the well-dispersed compounds (Fe_3_O_4_ and Gentamicin sulfate) in the coating which results in the increasing tortuosity of the oxygen and water vapor diffusion pathways. It is known that there is a need for sufficient H_2_O and O_2_ for the rust formation and dissolution of titanium for causing corrosion. If any of these processes are prevented, the corrosion is inhibited, and deposited coatings become efficient for corrosion prevention. Therefore, it is reasonable to believe that increasing the tortuosity of the diffusion pathways could prevent H_2_O and O_2_ from accessing the substrate surface, thus, leading to good anticorrosion properties [76].

The PANI-LIG:SG coatings exhibited the best anti-corrosion capability as evidenced by the highest and lowest values of Ecorr and Icorr (which corresponds to a lower corrosion rate).

Similar results of electrochemical behavior are found in literature and could be related to the reduction reaction of the polymeric matrix, which contributes in increasing the cathodic currents with corrosion rates of between 0.004 and 15 mm/year [42,77].

### 3.2. Biological Investigations

#### 3.2.1. In Vitro Cytotoxicity Evaluation

Morphological investigations were performed for osteoblast-like cells cultured during 48 h in contact with PANI-LIG- based thin coatings, to reveal information on the ability of these substrates for cell attachment and sustained growth. In these regards, fluorescence microscopy imaging was performed to evaluate the cytoskeleton appearance by specific staining of the actin filaments with Texas-Red–phalloidin.

**Figure 7:**
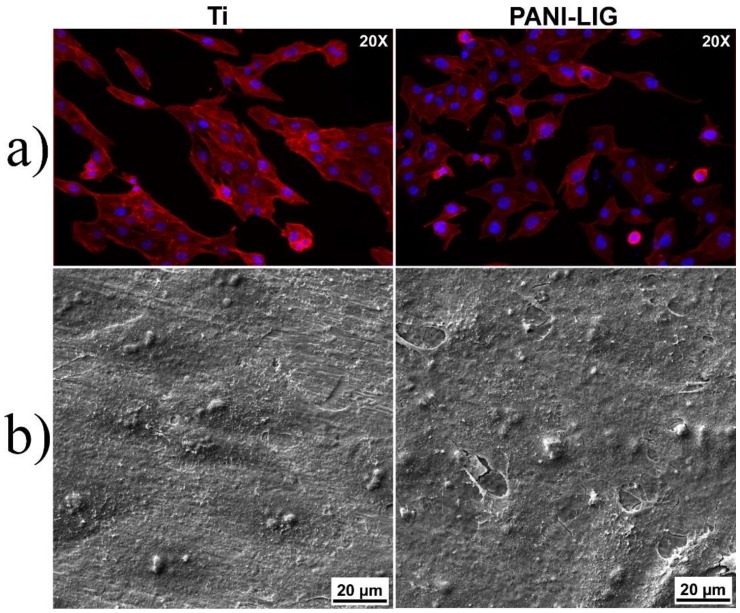
MG-63 cells fluorescence microscopy for substrate and PANI-LIG coatings (red Phalloidin & Hoechst staining) (**a**) and SEM images of MG-63 cells treated in the presence for substrate and PANI-LIG coatings at 48 h of incubation (**b**). (For interpretation of the references to color in this Figure legend, the reader is referred to the web version of this article.).

**Figure 8:**
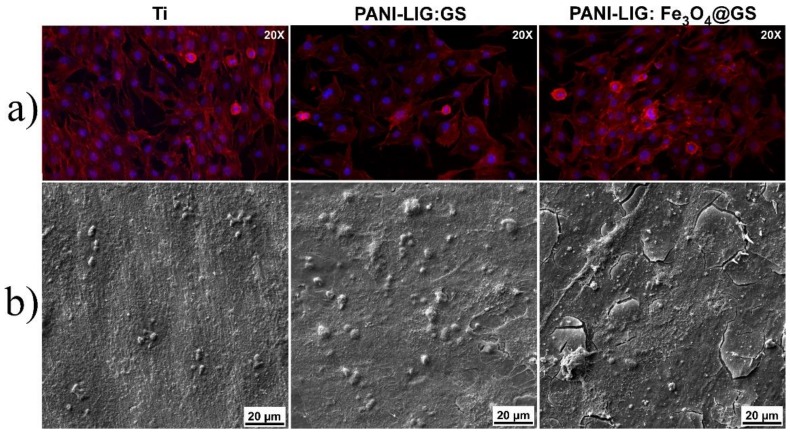
MG-63 cells fluorescence microscopy for substrate and PANI-LIG:GS and PANI-LIG:Fe_3_O_4_@GS coatings, respectively. (red Phalloidin and Hoechst staining) (**a**) and SEM images of MG-63 cells treated in the presence for substrate and PANI-LIG:GS and PANI-LIG:Fe_3_O_4_@GS coatings at 48 h of incubation (**b**). (For interpretation of the references to color in this Figure legend, the reader is referred to the web version of this article.).

Considered a marker of the cell’s well being, the cytoskeleton has many functions besides offering the shape of the cell [78]. For all deposited coatings, one can observe a good adhesion of the osteoblast-like cells, with a significantly uniform distribution spread (Figure 7 and Figure 8).

From the morphological point of view, the osteoblast-like phenotype with preferential flattened morphology was maintained for every deposited sample. Extensions of the actin filaments, the phylopodias, are strongly emphasized and would favor and strengthen the cells’ anchorage onto the coating surfaces. Moreover, specific longitudinal disposal of actin microfilaments and their multiple extensions, with eccentric prominent nuclei [68] was observed. This aspect was maintained in PANI-LIG samples as compared to control samples (Ti) (Figure 7).

In addition, the normal morphology of Hoechst- stained nuclei (blue), indicated that the compounds did not interfere with the normal growth of the existing cells [68]. One can observe distinct protuberances associated within the nucleus area of the individual cells, specifically delimiting the oval-shaped nucleoli within the cell nucleus [71].

Scanning electron microscopy investigations confirmed the information obtained from immunofluorescence evaluation of the cytoskeleton and nuclei morphology. Such behavior could be related to favorable interaction of the cells at the interface with the substrates. This is supported by the abundance of branched actin filaments protuberances extending to form connections from one cell to other and with the coating substrates (Figure 7 and Figure 8a,b).

**Figure 9:**
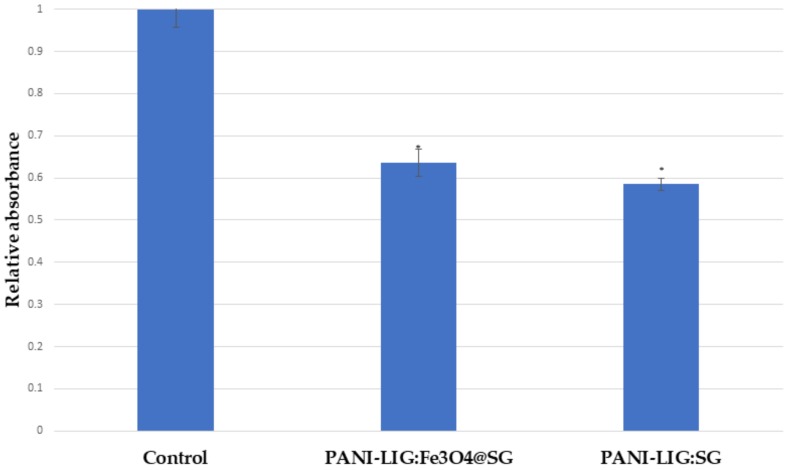
MG-63 cells metabolic activity for control (Ti), PANI-LIG:SG, and PANI-LIG:Fe3O4@SG coatings (data is expressed as relative to the Ti control; results are expressed as mean ± SD (*n* = 3) and presented as fold of control; student’s *t*-test *p* < 0.05 (*)).

However, in compliance with immunofluorescence and MTS viability measurements (Figure 9), the morphology of the MG-63 cells attached on GS- containing PANI-LIG coatings appeared slightly affected, as they seemed to possess a constricted shape leading to the conclusion that the cytoplasmic extensions might be providing cell–cell interactions, in spite of cell–substrate interactions [68]. Similar results on PANI-based coatings cytotoxicity behavior were shown on different cells: 3T3 fibroblasts [79]), PC-12 pheochromocytoma [80], and H9c2 cardiac myoblasts [81].

A change in cells’ behavior might be induced not by the deposited composite coatings, but the interaction of the osteoblast-like cells with the inorganic nanoparticles [42,82]. Moreover, supplementary investigations on the amount of antibiotic loaded into the samples can be made, as this might also influence the normal in vitro behavior of osteoblast-like cells.

#### 3.2.2. Anti-Biofilm Assay

The antimicrobial properties of conductive functionalized polyanilines have been investigated by exploring their interaction with microbial cells. It seems that acidic dopants on the polymer molecular chains react (by electrostatic carry charges of different polarity) with the microbial surface structures and induce membrane and cellular wall disruption resulting in cellular death in a time-exposure manner [83]. Data related to lower PANI concentrations which strongly inhibited the growth of several antibiotic resistant clinical pathogens and also laboratory strains of *Escherichia coli*, *Staphylococcus aureus* or *Pseudomonas aeruginosa* were found in the literature [26].

**Figure 10:**
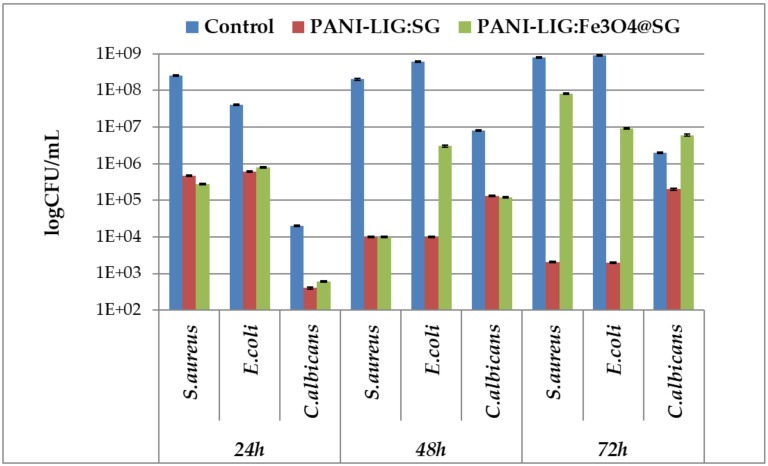
Colony-forming units (CFU)/mL values for *Staphylococcus aureus*, *Escherichia coli*, and *Candida albicans* biofilms after 24, 48, and 72 h of incubation in the presence of control (bare substrates) and MAPLE nano-modified coatings.

Our intention was to maximize the cumulative effect of the antibiotic by its combination with natural and renewable lignin, proved to be an important source of natural antimicrobial compounds [72,84]. Biofilm development assay demonstrated that the obtained coating has an increased inhibitory effect, especially against the tested bacteria species (i.e., *S. aureus* and *E. coli*) (Figure 10). The inhibitory effects were correlated with the exposure time, biofilms being more efficiently inhibited in early development stages (i.e., after 24 h of incubation). At the first two assessed time intervals (24 and 48 h), a significant biofilm inhibition can also be observed in the case of *C. albicans* tested strain, this phenotype is most probably explained by the use of the PANI-LIG surface, and the use of functional magnetite nanoparticles making an insignificant difference in the expressed antimicrobial effect of the coating (Figure 10).

Additionally, the simple antibiotic coatings proved to be a sustained antimicrobial activity, with CFU/mL values decreasing continuously on increasing the incubation time.

However, the antibiotic-containing coating begins to lose its efficiency in biofilm inhibition, especially in *E. coli*, commencing at the 48 h incubation time interval.

Overall, the biological assays demonstrated that the composite coatings synthesized by MAPLE can provide an efficient protection against microbial biofilms development, without inducing any cytotoxicity towards tested MG-63 cells. A more significant reduction in the number of viable microbial cells (compared to control) was observed for simple antibiotic coatings and a compromise must be made in choosing the optimum material which fulfills the vital concerns regarding the desired application (biocompatibility and antimicrobial efficiency).

## 4. Conclusions

By gathering the FTIR data (which indicate the preservation of functional groups integrity in the samples), the morphological topographical and wettability results (which emphasize the specific compounds rearrangements onto the substrates to form uniform and compact coatings, with local hill-like topography and hydrophilic behavior) one may conclude that all PANI-LIG based coatings were successfully MAPLE transferred by laser processing.

All coatings present the specific bands of studied materials, results also validated by XRD, which exhibit the poor crystalline nature of the coatings, with low-intensity maxima emerging corresponding to the base compounds. The effective functionalization of PANI-LIG coatings was proved, being confirmed by good dispersion of the drug and magnetite nanoparticles within the polymer matrix.

The antioxidant capacity of PANI-LIG based coatings has significant implications for their inclusion as biomaterials in biological media, with the PANI-LIG:GS coating proving to be the most efficient against corrosion, its Icorr being, in this case, 3.7 times smaller than for bare Ti, while its Ecorr increased 2.31 times. Our studies of cytotoxicity showed that PANI-LIG coatings might support osteoblast-like cell growth.

The most efficient antimicrobial activity, considered as long-term protection against adhesion and biofilms developed by *S. aureus*, *E. coli*, and *C. albicans* model strains, studied at three incubation time intervals, was assigned to PANI-LIG:GS samples. Corroborating the cytotoxicity and the antimicrobial activity measurements, along with the physico–chemical characterizations, we may conclude that the proposed conductive polymer-based coatings, either simple antibiotic or magnetite–antibiotic functionalized ones, have the potential for innovative systems for controlled drug release in an external-activated electric or magnetic field. The choose between them depends on the desired application. Nevertheless, further studies may, therefore, be envisioned to specifically evaluate the antibiotic release in electric and magnetic fields.

## Figures and Tables

**Table 1:** The roughness parameters values for polyaniline grafted lignin (PANI–LIG)-based coatings.

Sample	RMS (nm)	Ra (nm)	Rsk	Rku
PANI-LIG	134	98	0.942	8.122
PANI-LIG:GS	137.4	91.8	1.657	13.173
PANI-LIG:Fe_3_O_4_@GS	80.75	58.37	1.174	8.311

**Table 2:** The contact angle (CA) values for PANI-LIG based coatings.

Sample	Average Contact Angle Values [Degree]	Water Drop Evolution in Time [Degree]
PANI-LIG	67.8 ± 2.8	57.5 ± 1.4
PANI-LIG:GS	49.5 ± 0.9	48.6 ± 1.0
PANI-LIG:Fe_3_O_4_@GS	35.9 ± 2.0	34.2 ± 0.3
Bare Ti substrate	69.5 ± 4.5	68.9 ± 0.6

**Table 3:** Corrosion parameters (data extracted from the polarization curves) for bare Ti, PANI-LIG:Gentamicin sulfate (GS) and PANI-LIG:Fe_3_O_4_@GS coatings deposited on Ti.

Type of Coating	Ecorr vs. Ag/AgCl 3M, mV	Icorr, µA/cm^2^	Corrosion Rate (CR), mm/year	Rp, kΩ.cm^2^
Reference Ti	−257	0.074	0.64	322
PANI LIG:GS	−68	0.032	0.28	394
PANI LIG:Fe_3_O_4_@GS	55	0.053	0.46	290

## References

[1] Balint R., Cassidy N.J., Cartmell S.H. (2014). Conductive Polymers: Towards a Smart Biomaterial for Tissue Engineering. Acta Biomater..

[2] Ning C., Zhou Z., Tan G., Zhu Y., Mao C. (2018). Electroactive polymers for tissue regeneration: Developments and perspectives. Prog. Polym. Sci..

[3] Sima F., Ristoscu C., Duta L., Gallet O., Anselme K., Mihailescu I.N., Vilar R. (2016). Laser thin films deposition and characterization for biomedical applications. Laser Surface Modification of Biomaterials, Techniques and Applications.

[4] Popescu-Pelin G., Ristoscu C., Mihailescu I.N., Yang D. (2016). Laser ablation of biomaterials. Applications of Laser Ablation—Thin Film Deposition, Nanomaterial Synthesis and Surface Modification.

[5] Ringeisen B.R., Callahan J., Wu P.K., Pique A., Spargo B., McGill R.A., Bucaro M., Kim H., Bubb D.M., Chrisey D.B. (2001). Novel Laser-Based Deposition of Active Protein Thin Films. Langmuir.

[6] Cristescu R., Mihailescu I.N., Jelínek M., Chrisey D.B., Kassing R., Petkov P., Kulisch W., Popov C. (2006). Functionalized thin films and structures obtained by novel laser processing issues. Functional Properties of Nanostructured Materials.

[7] Pique A., Eason R. (2007). Deposition of Polymers and Biomaterials Using the Matrix-Assisted Pulsed Evaporation (MAPLE) Process. Pulsed Laser Deposition of Thin Films: Applications-Led Growth of Functional Materials.

[8] Chrisey D.B., Piqué A., McGill R.A., Horwitz J.S., Ringeisen B.R., Bubb D.M., Wu P.K. (2003). Laser Deposition of Polymer and Biomaterial Films. Chem. Rev..

[9] Kotwal A., Schmidt C.E. (2001). Electrical stimulation alters protein adsorption and nerve cell interactions with electrically conducting biomaterials. Biomaterials.

[10] Lee J.Y., Bashur C.A., Goldstein A.S., Schmidt C.E. (2009). Polypyrrole-coated electrospun PLGA nanofibers for neural tissue applications. Biomaterials.

[11] Wallace G.G., Smyth M., Zhao H. (1999). Conducting electroactive polymer-based biosensors. Trends Anal. Chem..

[12] Guo S.J., Li D., Zhang L.X., Li J., Wang E.K. (2009). Monodisperse mesoporous superparamagnetic single-crystal magnetite nanoparticles for drug delivery. Biomaterials.

[13] Lin Y.S., Wu S.H., Hung Y., Chou Y.H., Chang C., Lin M.L., Tsai C.P., Mou C.Y. (2006). Multifunctional Composite Nanoparticles: Magnetic, Luminescent, and Mesoporous. Chem. Mater..

[14] Lee J.E., Lee N., Kim H., Kim J., Choi S.H., Kim J.H., Kim T., Song I.C., Park S.P., Moon W.K. (2010). Uniform Mesoporous Dye-Doped Silica Nanoparticles Decorated with Multiple Magnetite Nanocrystals for Simultaneous Enhanced Magnetic Resonance Imaging, Fluorescence Imaging, and Drug Delivery. Am. Chem. Soc..

[15] Xuan S., Wang F., Lai J.M.Y., Sham K.W.Y., Wang Y.-X.J., Lee S.-F., Yu J.C., Cheng C.H.K., Leung K.C.-F. (2011). Synthesis of Biocompatible, Mesoporous Fe_3_O_4_ Nano/Microspheres with Large Surface Area for Magnetic Resonance Imaging and Therapeutic Applications. ACS Appl. Mater. Interfaces.

[16] Kim J., Kim H.S., Lee N., Kim T., Kim H., Yu T., Song I.C., Moon W.K., Hyeon T. (2008). Multifunctional uniform nanoparticles composed of a magnetite nanocrystal core and a mesoporous silica shell for magnetic resonance and fluorescence imaging and for drug delivery. Angew. Chem. Int. Ed..

[17] Piao Y., Kim J., Na H.B., Kim D., Baek J.S., Ko M.K., Lee J.H., Shokouhimehr M., Hyeon T. (2008). Wrap-bake-peel process for nanostructural transformation from beta-Feooh nanorods to biocompatible iron oxide nanocapsules. Nat. Mater..

[18] Lindig B.A., Rodgers M.A.J., Schaap A.P. (1980). Determination of the lifetime of singlet oxygen in water-d2 using 9,10-anthracenedipropionic acid, a water-soluble probe. J. Am. Chem. Soc..

[19] Nasongkla N., Bey E., Ren J., Ai H., Khemtong C., Guthi J.S., Chin S.F., Sherry A.D., Boothman D.A., Gao J. (2006). Multifunctional polymeric micelles as cancer-targeted, MRI-ultrasensitive drug delivery systems. Nano Lett..

[20] Rieter W.J., Kim J.S., Taylor K.M.L., An H., Lin W., Tarrant T., Lin W. (2007). Hybrid silica nanoparticles for multimodal imaging. Angew. Chem. Int. Ed..

[21] Liong M., Lu J., Kovochich M., Xia T., Ruehm S.G., Nel A.E., Tamanoi F., Zink J.I. (2008). Multifunctional inorganic nanoparticles for imaging, targeting, and drug delivery. ACS Nano.

[22] Van Schooneveld M.M., Vucic E., Koole R., Zhou Y., Stocks J., Cormode D.P., Tang C.Y., Gordon R.E., Nicolay K., Meijerink A. (2008). Improved Biocompatibility and Pharmacokinetics of Silica Nanoparticles by Means of a Lipid Coating: A Multimodality Investigation. Nano Lett..

[23] Kim J., Piao Y., Hyeon T. (2009). Multifunctional nanostructured materials for multimodal imaging, and simultaneous imaging and therapy. Chem. Soc. Rev..

[24] Cheng K., Peng S., Xu C.J., Sun S.H. (2009). Porous hollow Fe_3_O_4_ nanoparticles for targeted delivery and controlled release of cisplatin. J. Am. Chem. Soc..

[25] Belaabed B., Wojkiewicz J.L., Lamouri S., El Kamchi N., Lasri T. (2012). Synthesis and characterization of hybrid conducting composites based on polyaniline/magnetite fillers with improved microwave absorption properties. J. Alloys Compd..

[26] Grumezescu V., Andronescu E., Holban A.M., Mogoantă L., Mogoşanu G.D., Grumezescu A.M., Stănculescu A., Socol G., Iordache F., Maniu H. (2015). MAPLE fabrication of thin films based on kanamycin functionalized magnetite nanoparticles with anti-pathogenic properties. Appl. Surf. Sci..

[27] Metz S., Lohr S., Settles M., Beer A., Woertler K., Rummeny E.J., Daldrup-Link H.E. (2006). Ferumoxtran-10-enhanced MR imaging of the bone marrow before and after conditioning therapy in patients with non-Hodgkin lymphomas. Eur. Radiol..

[28] Wang Y.X., Hussain S.M., Krestin G.P. (2001). Superparamagnetic iron oxide contrast agents: Physicochemical characteristics and applications in MR imaging. Eur. Radiol..

[29] Ji J., Hao S., Wu D., Huang R., Xu Y. (2011). Preparation, characterization and in vitro release of chitosan nanoparticles loaded with gentamicin and salicylic acid. Carbohydr. Polym..

[30] Chang H.-I., Perrie Y., Coombes A.G.A. (2006). Delivery of the antibiotic gentamicin sulphate from precipitation cast matrices of polycaprolactone. J. Control. Release.

[31] Sionkowska A., Kaczmarek B., Gadzala-Kopciuch R. (2016). Gentamicin release from chitosan and collagen composites. J. Drug Deliv. Sci. Technol..

[32] Amezcua R., Friendship R.M., Dewey C.E., Gyles C., Fairbrother J.M. (2002). Presentation of postweaning Escherichia coli diarrhea in southern Ontario, prevalence of hemolytic E. coli serogroups involved, and their antimicrobial resistance patterns. Can. J. Vet. Res..

[33] Sarabia-Sainz A., Montfort G.R.C., Lizardi-Mendoza J., Sánchez-Saavedra M.D.P., Candia-Plata M.D.C., Guzman R.Z., Lucero-Acuña A., Vazquez-Moreno L. (2012). Formulation and characterization of gentamicin-loaded albumin microspheres as a potential drug carrier for the treatment of E. coli K88 infections. Int. J. Drug Deliv..

[34] Mihaiescu D.E., Cristescu R., Dorcioman G., Popescu C.E., Nita C., Socol G., Mihailescu I.N., Grumezescu A.M., Tamas D., Enculescu M. (2013). Functionalized magnetite silica thin films fabricated by MAPLE with antibiofilm properties. Biofabrication.

[35] Grumezescu A.M., Cristescu R., Chifiriuc M.C., Dorcioman G., Socol G., Mihailescu I.N., Mihaiescu D.E., Ficai A., Vasile O.R., Enculescu M. (2015). Fabrication of magnetite-based core–shell coated nanoparticles with antibacterial properties. Biofabrication.

[36] Chifiriuc M., Grumezescu A.M., Andronescu E., Ficai A., Cotar A.I., Grumezescu V., Bezirtzoglou E., Lazar V., Radulescu R. (2013). Water dispersible magnetite nanoparticles influence the efficacy of antibiotics against planktonic and biofilm embedded enterococcus faecalis cells. Anaerobe.

[37] Rașoga O., Sima L., Chirițoiu M., Popescu-Pelin G., Fufă O., Grumezescu V., Socol M., Stănculescu A., Zgură I., Socol G. (2017). Biocomposite coatings based on poly(3-hydroxybutyrate-co-3-hydroxyvalerate)/calcium phosphates obtained by MAPLE for bone tissue engineering. Appl. Surf. Sci..

[38] Kokubo T., Kushitani H., Sakka S., Kitsugi T., Yamamuro T. (1990). Solutions able to reproduce in vivo surface-structure changes in bioactive glass-ceramic A-W. J. Biomed. Mater. Res..

[39] Mihailescu M., Popescu R.C., Matei A., Acasandrei A., Paun I.A., Dinescu M. (2014). Investigation of osteoblast cells behavior in polymeric 3D micropatterned scaffolds using digital holographic microscopy. Appl. Opt..

[40] Rădulescu M., Holban A.M., Mogoantă L., Bălşeanu T.-A., Mogoșanu G.D., Savu D., Popescu R.C., Fufă O., Grumezescu A.M., Bezirtzoglou E. (2016). Fabrication, characterization, and evaluation of bionanocomposites based on natural polymers and antibiotics for wound healing applications. Molecules.

[41] Malich G., Markovic B., Winder C. (1997). The sensitivity and specificity of the MTS tetrazolium assay for detecting the in vitro cytotoxicity of 20 chemicals using human cell lines. Toxicology.

[42] Popescu-Pelin G., Fufă O., Popescu R.C., Savu D., Socol M., Zgură I., Holban A.M., Vasile B.Ş., Grumezescu V., Socol G. (2018). Lincomycin–embedded PANI–based coatings for biomedical applications. Appl. Surf. Sci..

[43] Paun I.A., Popescu R.C., Mustaciosu C.C., Zamfirescu M., Calin B.S., Mihailescu M., Dinescu M., Popescu A., Chioibasu D., Soproniy M. (2018). Laser direct writing by two-photon polymerization of 3D honeycomb-like structures for bone regeneration. Biofabrication.

[44] Grumezescu V., Holban A.M., Sima L.E., Chiritoiu M.B., Chiritoiu G.N., Grumezescu A.M., Ivan L., Safciuc F., Antohe F., Florica C. (2017). Laser deposition of poly(3-hydroxybutyric acid-co-3-hydroxyvaleric acid)—Lysozyme microspheres based coatings with anti-microbial properties. Int. J. Pharm..

[45] Cristescu R., Popescu C., Socol G., Visan A., Mihailescu I.N., Gittard S.D., Miller P.R., Martin T.N., Narayan R.J., Andronie A. (2011). Deposition of antibacterial of poly(1,3-bis-(p-carboxyphenoxy propane)-co-(sebacic anhydride)) 20:80/gentamicin sulfate composite coatings by MAPLE. Appl. Surf. Sci..

[46] Rădulescu M., Andronescu E., Dolete G., Popescu R.C., Fufă O., Chifiriuc M.C., Mogoantă L., Bălşeanu T.-A., Mogoşanu G.D., Grumezescu A.M. (2016). Silver nanocoatings for reducing the exogenous microbial colonization of wound dressings. Materials.

[47] Constantinescu C., Scarisoreanu N., Moldovan A., Dinescu M., Vasiliu C. (2007). Thin films of polyaniline deposited by MAPLE technique. Appl. Surf. Sci..

[48] Zu l., Cui X., Jiang Y., Hu Z., Lian H., Liu Y., Jin Y., Li Y., Wang X. (2015). Preparation and Electrochemical Characterization of Mesoporous Polyaniline-Silica Nanocomposites as an Electrode Material for Pseudocapacitors. Materials.

[49] Wu W., Pan D., Li Y., Zhao G., Jing L., Chen S. (2015). Facile fabrication of polyaniline nanotubes using the self-assembly behavior based on the hydrogen bonding: A mechanistic study and application in high-performance electrochemical supercapacitor electrode. Electrochim. Acta.

[50] Dhivya C., Anbu Anjugam Vandarkuzhali S., Radha N. (2015). Antimicrobial activities of nanostructured polyanilines doped with aromatic nitro compounds. Arab. J. Chem..

[51] Khalil M.I. (2015). Co-precipitation in aqueous solution synthesis of magnetite nanoparticles using iron (III) salts as precursors. Arab. J. Chem..

[52] Cornell R.M., Schwertmann U. (2003). The Iron Oxides. Structure, Properties, Reactions, Occurrences and Uses.

[53] Xu Z., Shen C., Tian Y., Shi X.Z., Gao H.J. (2010). Organic phase synthesis of monodisperse iron oxide nanocrystals using iron chloride as precursor. Nanoscale.

[54] Parveen N., Mahato N., Ansari M.O., Cho M.H. (2015). Enhanced electrochemical behavior and hydrophobicity of crystalline polyaniline@graphene nanocomposite synthesized at elevated temperature. Compos. Part B Eng..

[55] Zheng L., Su W., Qi Z., Xu Y., Zhou M. (2011). First-order metal-insulator transition and infrared identification of shape-controlled magnetite nanocrystals. Nanotechnology.

[56] Rowan A.D., Patterson C.H. (2009). Hybrid density functional theory applied to magnetite: Crystal structure, charge order, and phonons. Phys. Rev. B.

[57] Guo Y., Zhou Y. (2007). Polyaniline nanofibres fabricated by electrochemical polymerization: A mechanistic study. Eur. Polym. J..

[58] Sedenkove I., Trchova M., Blinova N.V., Stejskal J. (2006). In-situ polymerised polyaniline films. Preparation in solutions of hydrochloric, sulphuric or phosphoric acid. Thin Solid Film.

[59] Zhang L. (2007). The electrocatalytic oxidation of ascorbic acid on polyaniline film synthesized in the presence of β-naphthalenesulfonic acid. Electrochim. Acta.

[60] Palanikumar S., Meenarathi B., Anbarasan R. Synthesis, Characterization and applications of Gentamicin functionalized Fe_3_O_4_ nano hybrid. Proceedings of the ICRTIET-2014 Conference Proceeding.

[61] Bui T.Q., Ton S.N.-C., Duong A.T., Tran H.T. (2018). Size-dependent magnetic responsiveness of magnetite nanoparticles synthesised by co-precipitation and solvothermal methods. J. Sci. Adv. Mater. Devices.

[62] Ficai D., Ficai A., Vasile B.S., Ficai M., Oprea O., Guran C., Andronescu E. (2011). Synthesis of rod-like magnetite by using low magnetic field. Dig. J. Nanomater. Biostruct..

[63] Grumezescu A.M., Andronescu E., Holban A.M., Ficai A., Ficai D., Voicu G., Grumezescu V., Balaure P.C., Chifiriuc C.M. (2013). Water dispersible cross-linked magnetic chitosan beads for increasing the antimicrobial efficiency of aminoglycoside antibiotics. Int. J. Pharm..

[64] Elsayed A.H., Mohy Eldin M.S., Elsyed A.M., Abo Elazm A.H., Younes E.M., Motaweh H.A. (2011). Synthesis and properties of polyaniline/ferrites nanocomposites. Int. J. Electrochem. Sci..

[65] Rafienia M., Zarinmehr B., Poursamar S.A., Bonakdar S., Ghavami M., Janmalek M. (2012). Coated urinary catheter by PEG/PVA/gentamicin with drug delivery capability against hospital infection. Iran. Polym. J..

[66] Mateos-Timoneda M.A., Castano O., Planell J.A., Engel E. (2014). Effect of structure, topography and chemistry on fibroblast adhesion and morphology. J. Mater. Sci. Mater. Med..

[67] Blinova N.V., Stejskal J., Trchová M., Prokeš J. (2008). Control of polyaniline conductivity and contact angles by partial protonation. Polym. Int..

[68] Humpolicek P., Kasparkova V., Saha P., Stejskal J. (2012). Biocompatibility of polyaniline. Synth. Met..

[69] Bhattacharyya D., Xu H., Deshmukh R.R., Timmons R.B., Nguyen K.T. (2010). Surface chemistry and polymer film thickness effects on endothelial cell adhesion and proliferation. J. Biomed. Mater. Res. A.

[70] Ruardy T.G., Moorlag H.E., Schakenraad J.M., Van Der Mei H.C., Busscher H.J. (1997). Growth of Fibroblasts and Endothelial Cells on Wettability Gradient Surfaces. J. Colloid Interface Sci..

[71] Araujo J.R., Lopes E.S., de Castro R.K., Senna C.A., de Robertis E., Neves R.S., Fragneaud B., Nykänen A., Kuznetsov A., Archanjo B.S., Visakh P.M., Della Pina C., Falletta E. (2018). Characterization of Polyaniline-Based Blends, Composites, and Nanocomposites. Polyaniline Blends, Composites, and Nanocomposites.

[72] Nand A.V., Ray S., Easteal A.J., Waterhouse G.I.N., Gizdavic-Nikolaidis M., Cooney R.P., Kilmartin P.A. (2011). Factors affecting the radical scavenging activity of polyaniline. Synth. Met..

[73] Rodrigues P.C., Cantão M.P., Janissek P.R., Scarpa P.C.N., Mathias A.L., Pereira Ramos L., Gomes M.A.B. (2002). Polyaniline/lignin blends: FTIR, MEV and electrochemical characterization. Eur. Polym. J..

[74] Gizdavic-Nikolaidis M., Travas-Sejdic J., Bowmaker G.A., Cooney R.P., Kilmartin P.A. (2004). Conducting polymers as free radical scavengers. Synth. Met..

[75] Zhang W.L., Liu Y.D., Choi H.J. (2012). Fabrication of semiconducting graphene oxide/polyaniline composite particles and their electrorheological response under an applied electric field. Carbon.

[76] Chang C.-H., Huang T.-C., Peng C.-W., Yeh T.-C., Lu H.-I., Hung W.-I., Weng C.-J., Yang T.-I., Yeh J.-M. (2012). Novel anticorrosion coatings prepared from polyaniline/graphene composites. Carbon.

[77] Wang Y., Wang J., Zhang X. (2017). The electrochemical corrosion properties of PANI/coal composites on magnesium alloys. Int. J. Electrochem. Sci..

[78] Popescu R.C., Andronescu E., Vasile B.Ș., Truşcă R., Boldeiu A., Mogoantă L., Mogoșanu G.D., Temelie M., Radu M., Grumezescu A.M. (2017). Fabrication and Cytotoxicity of Gemcitabine-Functionalized Magnetite Nanoparticles. Molecules.

[79] Bayer C.L., Trenchard I.J., Peppas N.A. (2010). Analyzing polyaniline-poly(2-acrylamido-2-methylpropane sulfonic acid) biocompatibility with 3T3 fibroblasts. J. Biomater. Sci. Polym. Ed..

[80] Wang H., Ji L., Li D., Wang J.-Y. (2008). Characterization of nanostructure and cell compatibility of polyaniline films with different dopant acids. J. Phys. Chem. B.

[81] Bidez P.R., Li S., MacDiarmid A.G., Venancio E.C., Wei Y., Lelkes P.I. (2006). Polyaniline, an electroactive polymer, supports adhesion and proliferation of cardiac myoblasts. J. Biomater. Sci.-Polym. Ed..

[82] Ivashchenko O., Woźniak A., Coy E., Peplinska B., Gapinski J., Jurga S. (2017). Release and cytotoxicity studies of magnetite/Ag/antibiotic nanoparticles: An interdependent relationship. Colloid Surf. B.

[83] Pandiselvi K., Thambidurai S. (2015). Synthesis, characterization, and antimicrobial activity of chitosan–zinc oxide/polyaniline composites. Mater. Sci. Semicond. Process..

[84] Janković A., Eraković S., Ristoscu C., Serban N.M., Duta L., Visan A., Stan G.E., Popa A.C., Husanu M.A., Luculescu C.R. (2014). Structural and biological evaluation of lignin addition to simple and silver doped hydroxyapatite thin films synthesized by matrix-assisted pulsed laser evaporation. J. Mater. Sci. Mater. Med..

